# Population-based food consumption survey as an additional tool for foodborne outbreak investigations, Germany, 2017

**DOI:** 10.1017/S0950268820000564

**Published:** 2020-02-28

**Authors:** B. M. Rosner, A. Meinen, P. Schmich, M.-L. Zeisler, K. Stark

**Affiliations:** 1 Department for Infectious Disease Epidemiology, Robert Koch Institute, Berlin, Germany; 2 Department for Epidemiology and Health Monitoring, Robert Koch Institute, Berlin, Germany

**Keywords:** Foodborne infections, outbreaks

## Abstract

We conducted a food consumption survey in the general adult population of 18 years and older in Germany to obtain data on the frequency of consumption of food items that caused foodborne disease outbreaks in the past. A total of 1010 telephone interviews were completed that queried the consumption of 95 food items in the 7-day period before the interview. Survey results were weighted to be representative. Six exemplary ‘high risk’ food items were consumed by 6% to 16% of the general population. These were raw ground pork: 6.5%; ‘Teewurst’ (=spreadable sausage-containing raw pork): 15.7%; unpasteurised milk consumed without prior heating: 9.0%; food items prepared with raw eggs: 9.8%; unheated sprouts or seedlings: 8.8% and frozen berries consumed without prior heating: 6.2%. Data from our food consumption survey were comparable to data obtained from control persons in case-control studies conducted during past foodborne disease outbreak investigations. We consider our survey an additional helpful tool that will allow comparison with food consumption data from case-patients obtained in exploratory, hypothesis-generating interviews early on in outbreak investigations, and which may assist in forming hypotheses regarding associations of illnesses with suspected food vehicles. This may facilitate and accelerate investigations of future foodborne disease outbreaks.

## Introduction

About 370 foodborne disease outbreaks in Germany have been reported annually to the Robert Koch Institute between 2014 and 2018 through the routine surveillance system (SurvNet@rki). These outbreaks caused on average about 1700 reported gastrointestinal infections per year (range 1400–2100). However, because of under-ascertainment and under-reporting the actual number of foodborne disease outbreaks and associated illnesses in the population is assumed to be higher. A foodborne disease outbreak is defined as an ‘incidence, observed under given circumstances, of two or more human cases of the same disease and/or infection, or a situation in which the observed number of cases exceeds the expected number and where the cases are linked, or are probably linked, to the same food source’ [1]. On average, about 85% of the foodborne disease outbreaks that were reported in Germany were small (⩽5 cases), and about 50% occurred in private households. Typically, unless the disease is considered serious, e.g. botulism or haemolytic uraemic syndrome, these small outbreaks and outbreaks in single private households are not being investigated and the food item that caused the outbreak can only be guessed or suspected based on vague descriptive epidemiological evidence and often remains unknown. In contrast, larger or more serious foodborne disease outbreaks require a thorough outbreak investigation. The goal of the investigation is to identify the food item that caused the outbreak in a timely manner and to enable public health and food safety authorities to implement appropriate control measures that will stop the outbreak and prevent further illnesses.

Typically in the course of an outbreak investigation, in particular when cases are dispersed and a common place of exposure cannot be identified, case-patients are questioned extensively in exploratory interviews. The goal is to find some evidence for a common exposure, e.g. consumption of a certain food item in a defined time period before disease onset. The exploratory interviews may lead to one or more hypotheses regarding the association of certain food items with the occurrence of gastrointestinal infections in the outbreak. The hypotheses may then be tested in analytical epidemiological studies. In outbreaks where cases do not have an obvious common place of exposure, the analytical epidemiological study will often be designed as a case-control study where patients with the disease (cases) will be compared to a group of persons without the disease (controls) regarding the frequency of exposure to the suspected food items. However, it may be challenging to decide which hypotheses to test in an analytical epidemiological study. Consumed food items named frequently by case-patients in exploratory interviews may be food items that are consumed with high frequency in the general population as well and may not necessarily be causative for the infections in the outbreak. Therefore, it would be helpful to have data regarding the consumption of certain food items in the general population available for comparison with data from exploratory interviews. In contrast to other countries [2–4], to our knowledge, data on the consumption of food items in the general population, which could be used for comparison and hypotheses generation, do not exist in Germany. Food consumption surveys in Germany were conducted for other purposes, e.g. to survey the population's energy and nutritional intake. Questions in these surveys were not phrased in a way that is comparable to questions posed to case-patients in an outbreak investigation. For example, the queried time periods of possible exposure differ (24-h recalls or 4-week period *vs.* 7-day recall period in many foodborne disease outbreaks) [5–7]. Also, the questionnaires were not sufficiently detailed regarding consumption of food items that may play a role in foodborne disease outbreaks [7–9].

Therefore, we conducted a food consumption survey in a sample (about 1000 interviewees) of the general German speaking adult population (18 years and older) in Germany. The survey focused on the frequency of consumption of food items that caused foodborne disease outbreaks in Germany in the past (‘high risk’ food items) such as unheated sprouts and unpasteurised milk. We also queried food items that have been described in the literature as having caused foodborne disease outbreaks in other countries and may also be relevant for German consumers, e.g. certain raw vegetables. Finally, we included questions about the consumption of food items that were determined in population-based case-control studies conducted in Germany to be risk factors for certain bacterial or viral gastrointestinal infections, e.g. raw ground pork for yersiniosis [10]. To analyse if this data would have been helpful in previous foodborne disease outbreak investigations, we compared data from our food consumption survey to consumption data obtained in several exemplary, published outbreak investigations in Germany [11–17].

## Methods

### Study design

A representative sample of the German speaking general adult population (about 1000 persons aged at least 18 years) with residency in private households in Germany was interviewed over the telephone using a standardised questionnaire. Random sampling was conducted as described previously using a generated telephone sampling frame provided by ‘Arbeitskreis Deutscher Markt- und Sozialforschungsinstitute (ADM) [Working group of German market and social research institutes]’ [18, 19]. The sampling frame consisted of landline (60%) and cell phone numbers (40%) (dual-frame approach) in all 16 federal states in Germany. The telephone survey was designed in close collaboration between researchers at the Robert Koch Institute and experts at USUMA GmbH, a social research institute headquartered in Berlin, Germany, which was commissioned by the Robert Koch Institute for ad hoc telephone surveys [18]. The questionnaire was programmed in VOXCO Command Center™ Version 1.10.5 (Voxco, Montreal, CA), a software program for telephone interviews. Following pre-tests the questionnaire was shortened to the desired interview time frame of about 20 min. Interviews were conducted from 25 September to 14 November 2017 by trained interviewers of USUMA GmbH using computer-assisted telephone interviewing (CATI). When contacting households with several potential target persons via landline phone, the interviewee was randomly selected by CATI software based on the Kish selection grid. Thus, the likelihood of being interviewed was the same for all potential interviewees in the household [18, 20]. The response rate was calculated based on the criteria of the American Association for Public Opinion Research (AAPOR) using the AAPOR outcome rate calculator for dual-frame random-digit dialling (DFRDD), version 4.0, 2016 [21]. We report ‘response rate 3’ (RR3), which is the proportion of interviews conducted in relation to all probable households in the population [21]. The following formula was used:
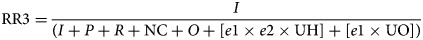
where *I* = complete interviews; *P* = partial interviews; *R* = refusal and break off with eligible case; NC = non-contact with eligible case; *O* = other non-interview with eligible case; UH = unknown if residential; UO = unknown other (residential, unknown if eligible) and *e* = the estimated proportion of cases of unknown eligibility that are eligible: *e*1 = the % of known-residential cases estimated to have eligible *R*, *e*2 = the % of unknown-if-residential cases that are estimated to be residential.

### Data protection

The study design was approved by the data protection officer of the Robert Koch Institute. Data protection measures included adherence to EU's general data protection regulation and a voluntary commitment to the guidelines of the ADM (Arbeitskreis Deutscher Markt- und Sozialforschungsinstitute) by USUMA GmbH [18, 22]. Participation in the telephone survey was voluntary. Interviews were conducted only if participants gave verbal consent to be interviewed after having been informed about the purpose of the study and data protection measures. Collected data were recorded anonymously.

### Questionnaire

The questionnaire queried consumption of certain food items within the 7-day period prior to the interview. The questionnaire was developed by scientists with expertise in foodborne disease outbreak investigations at the RKI and focused on food items that have the potential to cause foodborne outbreaks (‘high risk’ food items, e.g. raw ground meat; other kinds of meat; spreadable sausages that contain raw meat; certain fast food items; lettuce; mixed salads; raw vegetables; raw (unpasteurised) milk; raw milk cheeses; ice cream; eggs and egg products; smoked and other fish; salsa and other dips; fresh fruits; frozen berries; unpasteurised fruit juices; fresh herbs; dried tomatoes; herbal teas). Where plausible, certain food item variables were also operationalised. For example, raw ground pork, raw ground beef and raw ground mixed pork/beef, were combined to ‘any raw ground meat’. The questionnaire also queried information on general food consumption habits (vegetarian/vegan; certain food restrictions); supermarkets where food items had been purchased in the 14 days before the interview; some socio-demographic data (month and year of birth; sex; federal state of residency; county of residency; number of residents in village/town/city of residence; number of individuals living in the household; level of education) and some data that were required for the determination of weighting factors in the dual-frame design (e.g. number of landline phone numbers used in the interviewee household). Interviewees were also asked if they had travelled abroad in the 7 days before the interview and if their food consumption patterns in the 7-day time period before the interview had been atypical for them, e.g. due to an illness or a diet.

### Statistical analyses

Survey results were weighted to be representative of the German speaking general population of 18 years and older living in private households in Germany. An overall weighting factor was calculated that took the following aspects into account: the distribution of household sizes in the sample compared to the known distribution within the population of Germany (Micro Census; https://www-genesis.destatis.de/genesis/online); a design weighting factor reflecting the probabilities of selection as the target person for interviewing in the dual-frame study design; and an adjustment weighting factor correcting for deviations of the sample from the general population with respect to age group and sex, education and federal state of the place of residency. Data for adjustments, as of 31 December 2016, were obtained from the Federal Statistical Office (https://www-genesis.destatis.de/genesis/online).

Unweighted and weighted proportions with 95% confidence intervals (CIs) were calculated with Stata, version 15.1 (Stata Corporation, College Station, Texas, USA). Stratified analyses were conducted according to sex (male/female); age group (18–34 years, 35–64 years, 65 years and older); region of residency (North: residency in one of the following federal states: Bremen, Hamburg, Lower Saxony, Mecklenburg-West Pomerania, Schleswig-Holstein; East: Berlin, Brandenburg, Saxony, Saxony-Anhalt, Thuringia; West: Hesse, North Rhine-Westphalia, Rhineland-Palatinate, Saarland; South: Baden-Wuerttemberg, Bavaria); residency in urban *vs.* rural area (urban and rural as defined by the Federal Institute for Research on Building, Urban Affairs and Spatial Development) [23, 24] and level of education (level one (low or medium): no graduation from school or up to 10 years of general school education (up to ‘Hauptschulabschluss’ or ‘Realschulabschluss’); level two (high): more than 10 years of general school education (‘Abitur’ or ‘Fachhochschulreife’)). For the stratified analyses we picked six exemplary food items (raw ground pork; ‘Teewurst’ (=spreadable sausage-containing raw pork); unpasteurised (raw) milk; food items prepared with raw eggs; raw sprouts or seedlings and frozen berries) because they had caused foodborne disease outbreaks in Germany in the past, or because they had been identified as risk factors for bacterial or viral foodborne infections in population-based case-control studies conducted in Germany.

### Comparison with food consumption data obtained in previous outbreak investigation studies

To analyse if results from our food consumption survey would have been helpful in previous outbreak investigations we chose published examples of outbreak investigations that had been mainly conducted by the RKI, and where the food item that had caused the outbreak could be identified and was queried in our food consumption survey. Data regarding the frequency of consumption of this food item had been acquired from case-patients and a healthy comparison group (controls) in a case-control study [11–13, 16, 17], or from case-patients only [14, 15].

## Results

### Study population

A total of 1046 telephone interviews were completed, of which 1010 were included in the data analysis. Thirty six interviews were excluded because they were conducted as part of the pretest. In total, 50 453 telephone numbers were contacted, requiring 790 722 phone calls. The response rate (response rate 3 [21]) was 17.2% for landline telephone numbers and 16.2% for mobile telephone numbers. The overall response rate was 16.9%. On average, the telephone interview took 23 min. More women than men participated in the interviews (60% *vs.* 40%). The median age of participants was 59 years (range 18–95 years) (Table 1). About 5% (5.2%, 95% CI: 3.3–8.2) of study participants reported that they had travelled to another country (at least one overnight stay) in the 7 days before the interview, and about 16% (15.5%, 95% CI: 12.0–19.7) considered their food consumption pattern in the 7 days before the interview as atypical for them, when compared with a ‘normal’ week.

**Table 1.** Participants of the food consumption survey, Germany, 2017

Variable	Number of participants	%
Sex		
Female	602	59.6
Male	408	40.4
Age group (years)		
18–34	102	10.1
35–64	539	53.3
65 and older	369	36.5
Region of residency		
North	194	19.2
West	303	30.0
South	270	26.7
East	243	24.1
Area of residency		
Rural	340	33.7
Urban	670	66.3
Level of education		
Low-medium	506	50.2
High	501	49.8

Total number of participants (completed interviews) was 1010.

### Consumption of exemplary ‘high risk’ food items

The survey queried the consumption of 95 food items in the 7-day period before the interview (Table 2). Six exemplary ‘high risk’ food items were consumed by 6% to 16% of the general population. Raw ground pork was consumed by 6.5% (95% CI: 4.6–9.2) of the general population (Table 2). Raw ground pork tended to be consumed more frequently by men, persons 35–64 years of age, in eastern and northern parts of Germany and in rural regions, but 95% CIs between strata overlapped. The frequency of consumption of raw ground pork did not differ considerably between groups with high or low/medium level of school education (Table 3). ‘Teewurst’, a spreadable sausage-containing raw pork, was more frequently consumed by elderly persons (age group 65 years or older), in eastern regions of Germany, in rural areas and by persons with a low/medium level of school education. Unpasteurised milk was more frequently consumed by women, age groups up to 65 years, in the southern region, and in rural areas. Food items prepared with raw eggs, e.g. tiramisu, mayonnaise, were consumed more frequently by men, in the age group 18–34 years, in the northern region, and in rural areas. Persons of the age group 18–34 years, residents of urban regions and persons with a high level of education consumed unheated sprouts or seedlings more frequently compared with persons in the other strata of the respective category. Unheated frozen berries were consumed more frequently by women and in urban regions. Unheated frozen berries were least frequently consumed in the eastern region (Table 3).

**Table 2.** Consumption of food items in the general adult population (18 years and older) in the 7 days before the interview, Germany, 2017

Exposure in the 7 days prior to the interview	Weighted proportion (%)	95% CI
**Ground meat**		
Any ground mixed meat (pork and beef) (raw or cooked)	44.6	39.7–49.7
Raw ground mixed meat (pork and beef)	3.4	1.9–5.9
Any ground pork (raw or cooked)	13.5	10.6–17.0
Raw ground pork	6.5	4.6–9.2
Any ground beef (raw or cooked)	20.6	16.7–25.2
Raw ground beef	3.1	1.8–5.3
Any ground meat (mixed pork/beef or pork or beef)* (raw or cooked)	60.3	55.6–64.8
Raw ground meat (mixed pork/beef or pork or beef)*	10.2	7.7–13.5
**Other meat**		
Pork, e.g. filet, steak	48.4	43.6–53.3
Any pork (pork or ground pork or mixed (pork/beef) ground meat)*	69.6	65.1–73.7
Beef, e.g. filet, steak	40.1	35.3–45.0
Any beef (beef or ground beef or mixed (pork/beef) ground meat)*	71.3	67.0–75.1
Sheep meat, mutton or lamb meat	8.7	6.2–12.2
Wild boar meat or products made with wild boar meat, e.g. wild boar meat sausages	4.4	2.9–6.8
Game meat, e.g. venison, hare, pheasant	4.1	3.1–5.5
Chicken meat, e.g. chicken breast, boiling hen	63.5	58.8–68.0
Turkey meat, e.g. turkey breast, turkey filet	30.2	25.8–35.1
Duck meat	8.4	6.2–11.3
Goose meat	1.2	0.7–2.0
**Fast food**		
Doner kebab or chicken doner	17.1	13.3–21.7
Ready-to-eat sandwiches	14.5	11.0–18.9
**Sausage (cold cut) and spreadable sausages**		
Teewurst (spreadable sausage with raw pork)	15.7	12.2–19.8
Streichmettwurst (spreadable sausage with raw pork)	16.2	12.8–20.4
Zwiebelmettwurst (spreadable sausage with raw pork, contains onions)	12.6	9.3–16.8
Cervelatwurst (sausage with raw pork)	13.2	10.6–16.3
Leberwurst (spreadable sausage with liver)	39.6	34.8–44.5
Kalbsleberwurst (spreadable sausage with calf liver)	17.8	14.0–22.3
Leberwurst vom Schwein (spreadable sausage with pig liver)	21.2	17.6–25.4
Salami (from pig or cattle) or mini-salami	49.9	45.0–54.7
Salami (from poultry)	11.9	9.0–15.6
Landjäger, Räucherenden, Knacker or similar (dried, cured salami-style sausage with pork and/or beef)	17.6	14.1–21.9
Raw ham, Parma (Italian) ham, or Serrano (Spanish) ham (with pork)	45.3	40.5–50.1
**Hot dog style sausages**		
Hot dog style sausages, e.g. Frankfurter, Wiener	34.4	30.0–39.1
Hot dog style sausages, e.g. Frankfurter, Wiener; not heated before consumption	8.1	5.8–11.2
Hot dog style sausages, e.g. Frankfurter, Wiener; heated before consumption	29.0	24.9–33.4
**Lettuce, pre-cut packaged lettuce, ready-to-eat salad mix or similar**		
Lettuce, e.g. head lettuce, rucola	68.0	63.2–72.5
Pre-cut, packaged lettuce, e.g. bagged ready-to-eat lettuce	10.8	8.2–14.1
Ready-to-eat salad mix, e.g. lettuce with ham or grilled chicken	4.0	2.4–6.7
Pre-cut, packaged fruit or ready-to-eat, packaged fruit salad	3.7	2.0–6.7
**Raw (unheated) vegetables**		
Sprouts or seedlings, e.g. raw soy bean or mung bean sprouts, cress, radish sprouts or wheatgrass	8.8	6.4–11.8
Tomatoes	84.7	80.8–87.9
Cucumbers	72.8	68.0–77.1
Bell peppers	61.8	57.1–66.4
Carrots	52.2	47.2–57.1
Green onions/scallions or leek	33.2	28.8–37.9
Mushrooms	11.9	8.9–15.8
**Nuts or seeds, spreadable creams from nuts or seeds**		
Raw, unprocessed nuts or seeds, e.g. hazelnuts, almonds, pumpkin seeds or sesame seeds	51.1	46.2–55.9
Peanut butter, peanut cream or similar	6.3	4.2–9.3
Sesame butter, sesame cream or similar	0.8	0.4–1.6
Other butters/creams with nuts or seeds, e.g. almond butter, cashew butter	5.2	3.7–7.3
**Raw (unpasteurised) milk**		
Raw milk (also if mixed with chocolate powder)	11.5	8.4–15.5
Raw milk, consumed without prior heating	9.0	6.2–13.0
Raw milk, consumed with prior heating	2.4	1.4–4.2
Raw milk, purchased directly at a farm (‘Milch ab Hof’)	5.8	3.8–8.9
Raw milk, from specialised producers (‘Vorzugsmilch’)	5.7	3.5–9.0
Raw milk, purchased at a vending machine for raw milk (‘Milchtankstelle’)	0.8	0.4–1.7
**Ice cream**		
Ice cream purchased at an ice cream stand/store (also soft ice cream, frozen yogurt)	19.0	15.2–23.5
Industrially produced ice cream, e.g. packaged in containers from the frozen food section in a store	33.9	29.4–38.8
**Cheese**		
Cheese with white or blue mould, e.g. Camembert, Brie, Gorgonzola	43.3	38.6–48.2
Goat's milk cheese	16.7	13.4–20.5
Sheep's milk cheese, feta cheese	32.2	27.7–37.0
Mozzarella	36.9	32.2–41.8
Hard cheese, e.g. Appenzeller, Emmentaler, Parmesan	64.6	59.7–69.2
Quargel cheese or similar (Harzer Käse, Korbkäse or Handkäse)	15.0	12.1–18.5
Pre-cut, packaged cheese, e.g. slices of Gouda, or slices of various cheeses (‘Käseaufschnitt’)	62.7	57.9–67.4
**Eggs and egg products**		
Chicken eggs, e.g. as boiled egg, scrambled egg, fried egg (sunny side up) or omelette	81.4	77.2–84.9
Quail eggs	1.2	0.6–2.7
Unheated food items that were prepared with raw eggs, e.g. homemade mayonnaise, tiramisu, mousse, raw cake dough, ‘Stockbrotteig’ (dough for bread on a stick)	9.8	7.1–13.3
**Fish, shellfish, seafood**		
Salted herring (uncooked) (‘Matjeshering’, ‘Bismarckhering’ or similar)	21.3	17.9–25.2
Other fresh fish (whole fish or fish fillet, e.g. redfish or pollock)	33.5	29.1–38.2
Sushi with fish or shellfish	7.2	4.7–11.0
Oysters or other mussels	3.4	2.1–5.6
Other seafood, e.g. lobster, shrimp, squid	16.2	12.7–20.3
Smoked salmon (also pre-cut and packaged in plastic)	20.4	17.2–24.1
Other smoked or dried fish, e.g. trout, eel, mackerel (also packaged in plastic)	14.7	11.5–18.6
**Sauces and dips**		
Salsa from fresh tomatoes	6.7	4.5–9.9
Avocado cream or guacamole	10.7	8.1–14.1
Pesto sauce	14.6	11.4–18.6
**Fresh fruit or frozen berries**		
Apple	82.6	78.0–86.4
Pear	36.9	32.2–41.7
Water melon	8.5	6.2–11.4
Other melon, e.g. honeydew melon	11.8	8.9–15.6
Fresh strawberries	9.4	6.7–13.1
Fresh raspberries	16.7	13.7–20.2
Other fresh berries, e.g. blackberries, currants, gooseberries	15.9	12.9–19.5
Grapes	63.5	58.5–68.3
Fresh papaya or mango	15.6	12.5–19.4
Frozen berries, e.g. frozen strawberries, raspberries, or berry mix	9.9	7.7–12.6
Frozen berries, heated before consumption	3.1	2.0–4.6
Frozen berries, not heated before consumption	6.2	4.5–8.5
**Fruit or vegetable juices**		
Fruit shake or smoothie (also smoothie from squeezable plastic container)	9.9	7.3–13.4
Freshly pressed, unpasteurised apple juice (also mixed with water)	20.7	17.0–25.0
Other freshly pressed, unpasteurised fruit or vegetable juice (also mixed with water)	14.3	11.3–18.0
**Herbs**		
Fresh herbs, not heated before consumption, e.g. basil, chive, mint	59.8	54.9–64.5
**Specialty foods**		
Paste of chickpeas (hummus), paste of sesame (tahini or halwa)	8.8	6.3–12.1
Dried tomatoes	9.1	7.0–11.7
Vitamins or minerals as food supplements, e.g. vitamin C, iron, magnesium	29.9	25.5–34.7
Other food supplements, e.g. goji berries, spirulina, (also as powder, tablets or capsules)	3.2	2.1–5.0
**Sweets and desserts**		
Chocolate bar or candy bar with chocolate	60.3	55.4–65.1
Cake or pie, purchased (not homemade cake or pie)	31.0	26.7–35.7
Sweet pastries or pastries filled with cream (not homemade pastries)	17.0	13.8–20.8
Frozen pie or frozen pastries with berries	5.0	3.1–8.0
**Water and tea**		
Tap water (drunk without prior heating; not tap water used to prepare tea etc.)	58.4	53.5–63.1
Mineral water (in bottles or TetraPak® carton)	79.1	74.6–83.0
Herbal tea or fruit tea, e.g. mint tea, or fennel tea	64.9	60.2–69.4
Other tea, e.g. black tea or green tea (also flavoured tea)	41.0	36.3–45.9

Results from weighted analyses are shown. Variables with * were operationalised.

**Table 3.** Consumption of six exemplary ‘high risk’ food items (proportion (%) exposed) in subgroups of the general adult population (18 years and older), Germany, 2017

		Raw ground pork (%)	‘Teewurst’^a^ (%)	Unpasteurised milk (%)	Food items with raw eggs (%)	Unheated sprouts or seedlings (%)	Unheated frozen berries (%)
Sex	Male	9.6	16.6	7.2	12.3	8.7	4.2
	95% CI^b^	6.5–13.9	11.4–23.4	4.0–12.8	8.1–18.5	5.5–13.6	2.4–7.0
	Female	3.5	14.8	10.8	7.3	8.8	8.2
	95% CI	1.5–7.6	10.6–20.1	6.7–17.1	4.5–11.5	5.8–13.1	5.6–12.0
Age group	18–34 years	4.9	9.9	10.6	15.1	14.0	6.1
	95% CI	2.3–10.2	4.1–22.1	4.8–21.6	7.3–28.7	7.0–26.0	2.3–15.1
	35–64 years	8.2	13.2	10.6	7.9	9.1	6.9
	95% CI	5.0–13.1	8.8–19.4	6.5–16.6	5.3–11.6	6.5–12.5	4.7–10.1
	65 years and older	4.6	25.4	4.8	8.8	3.5	4.9
	95% CI	2.7–7.8	19.0–33.0	1.8–12.5	5.7–13.3	2.0–6.2	3.0–7.8
Region of residency	North	9.5	12.9	6.3	14.7	7.2	6.4
	95% CI	4.6–18.5	8.0–20.3	2.6–14.3	7.7–26.3	3.0–16.4	3.5–11.3
	West	4.4	17.3	7.3	7.3	8.8	7.7
	95% CI	2.3–8.2	11.0–26.3	3.6–14.4	4.2–12.5	5.4–14.0	4.8–12.3
	South	1.7	10.7	14.3	8.2	8.7	6.3
	95% CI	0.6–4.8	5.6–19.5	8.1–23.9	4.3–14.9	5.3–14.0	3.1–12.7
	East	15.1	23.5	6.6	11.8	10.3	3.0
	95% CI	8.7–24.9	15.7–33.5	2.2–18.2	5.7–22.9	4.5–21.9	1.6–5.6
Urban/rural residency	Urban	4.3	13.6	7.2	8.4	10.6	7.2
	95% CI	2.8–6.7	9.8–18.5	4.4–11.5	5.4–12.9	7.4–15.0	4.9–10.5
	Rural	10.8	19.9	12.8	12.5	5.0	4.2
	95% CI	6.4–17.8	13.5–28.2	7.2–21.7	7.9–19.3	3.1–8.0	2.6–6.9
Level of education	Low or medium	6.0	19.7	9.4	10.3	6.2	5.6
	95% CI	3.7–9.5	14.9–25.5	5.7–14.9	6.9–15.0	3.7–10.1	3.8–8.1
	High	7.2	7.0	8.5	8.8	14.4	7.7
	95% CI	4.2–12.0	4.7–10.2	5.1–13.8	5.2–14.6	10.0–20.4	4.4–13.0

Results from weighted analyses are shown. See Table 1 for the number of participants in each subgroup.

a Teewurst = spreadable sausage-containing raw pork.

b Confidence interval.

### Comparison with food consumption data obtained in previous outbreak investigations

We analysed if data from the food consumption survey would have been useful in previous outbreak investigations. The exemplary outbreaks had been caused by contaminated fenugreek sprouts (STEC O104:H4) [12], mung bean sprouts (*Salmonella* Newport) [13], ‘Teewurst’ (*Salmonella* Derby) [14] , raw ground pork and spreadable pork sausages (*Salmonella* Muenchen) [15] and raw ham (*Salmonella* Kottbus) [16, 17] (Table 4). Compared with the food consumption survey the frequency of consumption of the food items that caused the respective outbreak was substantially higher among the interviewed case-patients in the exemplary outbreaks. For instance, consumption of raw sprouts or seedlings was reported by 9% of the population in our food consumption survey. This was comparable to the proportion of control persons that reported sprout consumption in case-control studies during the investigation of the STEC O104:H4-outbreak in Germany, despite slight differences regarding the time period that was considered (7 days and 14 days before the interview, respectively). In contrast, 25% of case-patients remembered having consumed sprouts before disease onset [12]. The frequency of consumption of raw tomatoes (this survey: 85%), cucumbers (73%) and leaf lettuce (68%), food items that were suspected as causative infection vehicles early on in the outbreak investigation in 2011 [11], did not differ substantially between the general population and the case-patients (or the control persons) in the outbreak investigation (Table 4). The frequency of consumption of food items that caused the *Salmonella* Newport outbreak (sprouts) or the *Salmonella* Kottbus outbreak (raw ham) was considerably lower in the food consumption survey when compared with case-patients and very similar, or at least in about the same range, when compared with control persons (Table 4). In a *Salmonella* Derby outbreak and a *Salmonella* Muenchen outbreak where case-control studies were not conducted as part of the outbreak investigation [14, 15], the frequency of consumption of food items that caused the outbreak (‘Teewurst’, and raw ground pork/spreadable sausage with pork, respectively) was lower in the food consumption survey than among the interviewed case-patients. When we compared the frequency of consumption of ‘Teewurst’ of case-patients of the *Salmonella* Derby outbreak, which had mainly affected elderly persons, with the age group 65 years and older in the food consumption survey there was still a marked difference (70% *vs.* 25%).

**Table 4.** Comparison of food consumption data obtained by questioning case-patients and control persons in past outbreak investigations in Germany with data obtained in food consumption survey of the general adult population, Germany, 2017

Causative agent of the outbreak (year of outbreak; number of cases)	Causative (or suspected) food item	Consumption frequency among case-patients^a^ (%)	Consumption frequency among control group (%)	Consumption frequency in the general population (this study) (%)
Shiga toxin-producing *E. coli* (STEC) O104:H4 (2011; >3800 cases)	Sprouts	25	9	9^b^
	Raw tomatoes^c^	85	77	85
	Raw cucumbers^c^	88	66	73
	Leaf lettuce^c^	77	64	68
*Salmonella* Newport (2011; 106 cases)	Mung bean sprouts	33	2	9^b^
*Salmonella* Derby (2013/14; 145 cases, mainly elderly patients)	‘Teewurst’^d^	70	Not determined	16 (25^e^)
*Salmonella* Muenchen (2014; 247 cases)	Raw ground pork	27	Not determined	7
	Sausages that contain raw pork	24	Not determined	16
*Salmonella* Kottbus Cluster 1 (2017; 51 cases)	Raw ham	82	47	45

a Determined in hypothesis-generating interviews or in case-control studies.

b ‘Unheated sprouts or seedlings’.

c Food items that had been suspected early in the outbreak investigation.

d ‘Teewurst’: spreadable sausage that contains raw pork.

e Age group of 65 years and older.

## Discussion

Representative data regarding the consumption of a variety of food items that are known risk factors of bacterial and viral gastrointestinal infections or that were identified as infection vehicles in past foodborne disease outbreaks were generated for the population of 18 years and older in Germany for the first time with our food consumption survey.

Food items that could be considered to put consumers at an elevated risk of obtaining a gastrointestinal infection were consumed by 6% to 16% of the population, despite publicly available consumer recommendations that state their health risks. About 10% of the population ate raw ground meat (pork, beef or a mixture) within a 7-day time period. Raw ground pork, often mixed with spices and onions (known as ‘Mett’ or ‘Hackepeter’) on bread rolls (‘Mettbrötchen’), is a popular food item in Germany, especially in certain regions (Eastern and Northern Germany). Consumption of raw ground pork has been shown to be a risk factor for infections with *Yersinia enterocolitica* [10] and *Salmonella* Typhimurium [25] in Germany, and was associated with an outbreak of *Campylobacter coli* [26]. Consumption of raw or undercooked ground beef was associated with Shiga toxin-producing *Escherichia coli* (STEC) infections, albeit not statistically significantly [27]. Consumption of raw ground meat is discouraged by numerous consumer recommendations published by the Federal Institute for Risk Assessment (BfR) in Germany, especially with respect to vulnerable consumer groups, e.g. young children, the elderly, pregnant women and people with a compromised immune system, for example in [28–30].

Another ‘high risk’ food item, ‘Teewurst’, was consumed by 16% of the general population (over all age groups). The proportion of people in the age group ≥65 years that had consumed ‘Teewurst’ was even higher (25%). Consumer recommendations published by the BfR discourage consumption of spreadable sausages that contain raw meat. In particular, they advise against serving these food items to elderly persons and other vulnerable groups in nursing homes, hospitals or other community facilities [31]. An outbreak among mainly elderly persons, many of them in nursing homes or hospitals, caused by *Salmonella* Derby in Germany in 2013/2014 was associated with consumption of ‘Teewurst’, which indicated that the published recommendations may not have been known or may not have been taken seriously [14].

The proportion of people who reported having consumed unpasteurised milk (12%), in particular without prior heating (9%), was surprisingly high. In the United States and in Canada about 3% reported consumption of any unpasteurised milk [2, 3]. One explanation may be that some of our interviewees misclassified the type of consumed milk, even though a definition of ‘raw’ or unpasteurised milk had been provided in the interview. Unpasteurised milk that had not, or not adequately, been heated before consumption has caused numerous *Campylobacter jejuni* and several STEC outbreaks in Germany in the past few years [32–34]. The BfR states in consumer information material that consumption of unheated unpasteurised milk poses a threat to human health, and advises against its consumption especially by vulnerable persons such as young children, the elderly and persons with a compromised immune system [35]. Less than 1% of our interviewees reported having consumed raw milk obtained from a milk-vending machine at a farm. In the past few years, several *Campylobacter* outbreaks have been reported that were associated with milk-vending machines [32, 34]. Even though a label that recommends heating of the milk before consumption must be placed on milk-vending machines, this consumer advice is not being followed by all consumers who purchase this type of milk, or the label is missing on some vending machines.

About 9% of the population reported having eaten sprouts or seedlings within a 7-day time period that were not heated before consumption. This proportion is comparable to results obtained from control persons in case-control studies conducted during the STEC O104:H4 outbreak in Germany in 2011, but higher than results from the control group in a case-control study conducted in the investigation of a *Salmonella* Newport outbreak caused by mung bean sprouts later in the same year (2%). Sprouts and seedlings are considered so-called ‘stealth’ food items because they may be served mixed with other food items, e.g. salads, or as toppings and, therefore, their consumption is particularly difficult to recall [36]. In Canada, about 13% of the population reported consumption of any sprouts and in the United States, 4% to 8% reported sprout consumption, depending on the types of sprouts. In these food consumption surveys, it is not specified whether the sprouts were consumed cooked or uncooked [2, 3].

The main purpose of our survey was to have food consumption data available for future foodborne disease outbreak investigations. We used data from several past outbreak investigations to evaluate whether the food consumption data may have been helpful in these investigations. In all foodborne disease outbreaks listed in Table 4, results from the food consumption survey would have shown that consumption of the food item that ultimately caused the outbreak was substantially lower in the general population than in the group of case-patients. This information would have helped to direct the outbreak investigation towards the causative food vehicle earlier on in the investigations. In several unpublished outbreak investigations that have been conducted by our group since completion of our survey (*Salmonella* Mikawasima (2018); *Salmonella* Hadar (2019); hepatitis A virus (2019, still ongoing)), the food consumption data proved to be useful for hypothesis generation as well. In these outbreak investigations, initially suspected food items (cucumbers, ground meat and grapes, respectively) were considered as less likely to be the infection vehicle because they were consumed with similar frequency by case-patients and the general population. Unfortunately, the food items that caused these outbreaks could not be identified unequivocally.

The number of interviewees in our food consumption survey was much smaller than in food consumption telephone surveys that were conducted in the United States (17 372 interviewees) [2] or Canada (10 942 interviewees) [3], and our survey did not cover a 1-year period [2, 3]. There are some limitations to using data from our food consumption survey in outbreak investigations. The survey was conducted to be representative of the general German-speaking population of 18 years and older. Most likely, the data will not be very helpful in investigations of foodborne disease outbreaks among special groups of persons, for example, young children or the elderly, or if the outbreak is localised to a certain region of Germany, where food consumption patterns may differ from those of the general population. Also, we could only enquire about consumption of a limited number of food items in the survey and future outbreaks may involve food items that have not been queried in our food consumption survey. The consumption of certain food items, for example, fresh berries, likely varies by season. Our food consumption survey was deliberately conducted in the autumn (end of September to mid-November) because we considered this a time of the year, where there would be only little seasonality in food consumption, compared with, for example, the summer. We are aware that the survey should be repeated in other seasons of the year. The food consumption data may not be helpful either in the investigation of future outbreaks that will be caused by pathogens with a much longer or shorter incubation period than 7 days, the time frame we chose to question our interviewees about [37].

Recruitment of control persons for case-control studies in outbreak investigations is generally viewed as being time and resource intensive. Therefore, other methods are being developed in order to expand the toolbox for hypotheses generation and testing. Food consumption survey data were already used in outbreak investigations in other countries [38, 39]. Background exposure data from food consumption surveys can be applied for binomial probability analyses, a method that was developed at Oregon Health Authority [40]. Besides food consumption survey data, market share data [41], shopping card or till receipt information [42] and data from market-research panels [43–45] have been used in outbreak investigations. We consider our food consumption survey an additional helpful tool for outbreak investigations in Germany. It will allow comparison of food consumption data from case-patients obtained in exploratory, hypothesis-generating interviews with data from the general population early on in the outbreak investigation, and may support the generation of hypotheses regarding associations of illnesses with suspected food vehicles. We realise that this approach will not replace analytical epidemiological studies in future outbreak investigations, but food consumption data from the general population may allow us to limit our hypotheses to fewer suspected food items, which may facilitate and accelerate the outbreak investigation.
